# Curcumol ameliorates neuroinflammation after cerebral ischemia–reperfusion injury via affecting microglial polarization and Treg/Th17 balance through Nrf2/HO-1 and NF-κB signaling

**DOI:** 10.1038/s41420-024-02067-3

**Published:** 2024-06-24

**Authors:** Ying Liu, Wen Wang, Bohan Di, Jiangyong Miao

**Affiliations:** Department of Neurology, Second Hospital of Hebei Medical University, Shijiazhuang, 050000 Hebei China

**Keywords:** White matter injury, Neuroimmunology

## Abstract

Neuroinflammation caused by microglia and other immune cells plays pivotal role in cerebral ischemia/reperfusion injury and recovery. Modulating microglial polarization or Treg/Th17 balance from pro-inflammatory phenotype to anti-inflammatory phenotype are promising strategies for the treatment of cerebral ischemia. Curcumol has potential to fight against oxidative stress and inflammation, but whether it has protective effect in cerebral ischemia is uncertain. In the present study, cerebral ischemia was induced in C57BL/6 mice via middle cerebral artery occlusion (MCAO). MCAO mice were treated with curcumol for 7 days, then post-stroke ischemic injury, neurological deficits, microglial polarization and brain leukocyte infiltration were evaluated by TTC staining, behavioural tests, flow cytometry, western blot and immunofluorescence. We found that poststroke administration of curcumol reduced infarct volume, attenuated neuronal damage and inflammation, and improved motor function recovery of MCAO mice. Curcumol skewed microglial polarization toward anti-inflammatory phenotype in MCAO mice in vivo or after oxygen-glucose deprivation and reoxygenation (OGD/R) in vitro. In addition, curcumol reduced local T cell infiltration in ischemic brain of MCAO mice and impaired Treg/Th17 balance. Curcumol inhibited ROS production and regulated Nrf2/HO-1 and NF-κB signaling in microglia. Finally, inhibiting Nrf2/HO-1 signaling or activating NF-κB signaling abrogated the influence of curcumol on microglial polarization. In conclusion, curcumol treatment reduced brain damage and neuroinflammation via modulating anti-inflammatory microglial polarization and Treg/Th17 balance through Nrf2/HO-1 and NF-κB signaling. Curcumol might be a promising treatment strategy for stroke patients.

## Introduction

Stroke is caused by blockage of blood vessels due to thrombus or embolus. It ranks the second leading cause of death worldwide [1]. Stroke leads to permanent disabilities in 80% of its survivors [2]. It is estimated that there are 5.9 million deaths and 102 million disabilities caused by stroke every year all over the world [3]. The incidence of stroke is increasing due to demographic changes and increasing prevalence of obesity, diabetes mellitus, hypertension and hyperlipidemia [4]. Cerebra ischemia represents 87% of all stroke cases [5]. During cerebral ischemia, low oxygen supply and lack of nutrients lead to serious damage of ischemic brain. Treatments of cerebral ischemia are largely relying on recanalization strategies, but unfortunately reestablishing of blood flow also causes secondary brain damage due to increasing oxidative stress and neuroinflammation. Neuroinflammation, intimately associated with oxidative stress, significantly contributes to neuronal injury in the acute phase of cerebral ischemia by provoking death of cells and penetration of inflammatory cells in the ischemic region [6]. Nevertheless, neuroinflammatory pathways also show beneficial effects in the late phase of cerebral ischemia by promoting tissue repair and functional recovery [6]. Thus, elucidating the underlying molecular mechanisms of neuroinflammation and developing strategies to combat the pathological responses may potentiate therapeutic development of cerebral ischemia.

Microglia are tissue resident macrophages and the major immune cells in central nervous system. They can be rapidly activated after cerebral ischemia, and play an important role in brain damage, neuronal death and neuroinflammation [7]. Activated microglia can be polarized into two distinct phenotypes, namely, the classical M1 phenotype and the alternatively activated M2 phenotype in respond to microenvironmental changes. The classical M1 type microglia are proinflammatory and produce multiple proinflammatory mediators such as interleukin-1β (IL-1β), IL-6, iNOS and tumor necrosis factor α (TNF-α) [8]. In contrast, the alternatively activated M2 type microglia are anti-inflammatory and secrete anti-inflammatory cytokines such as IL-4, IL-10 and TGF-β. There are growing evidences indicating that activated microglia act a double-edged role in cerebra ischemia by either polarizing to M1 or M2 type [9]. The M1 type microglia exacerbate neuroinflammation and brain damage, while M2 type microglia facilitate neuron survival and tissue repair. Therefore, a shift of microglia from M1 pro-inflammatory type to M2 anti-inflammatory type may be a promising therapeutic strategy for cerebral ischemia.

During cerebral ischemia, the blood-brain barrier breaks, thus blood-borne immune cells such as neutrophils, macrophages and T cells can infiltrate into the ischemic brain. Among them, T cells take part in the later phase of cerebral ischemia. T helper type 17 (Th17) cells and regulatory T cells (Tregs) are derived from a common precursor cell (naive T cell) and require the same signal pathway (TGF-β signaling) to initiate differentiation [10]. However, Th17 cells and Tregs have opposite functions. Th17 cells trigger inflammation and cause autoimmunity, while Tregs suppress inflammation and maintain immune homeostasis [11]. Emerging evidences suggest that Th17 cells and Tregs are involved in poststroke inflammation, brain injury and tissue repair [12]. For example, Liesz et al. find that depletion of Tregs profoundly augments poststroke activation of resident microglia and invading T cells, thus increases brain damage and inhibits functional recovery [13]. In contrast, increasing accumulation of Th17 cells in ischemic brain is associated with worsen neuroinflammation and stroke outcome [14]. Thus, modulating Treg/Th17 balance may attenuate neuroinflammation and cerebral ischemia injury to some extent.

Natural products have generated an excellent reservoir for developing new drugs of various diseases, including cerebral ischemia [15]. Curcumol, a bioactive sesquiterpenoid, has been isolated from various plants from Zingiberaceae. In recent years, curcumol attracts much attention due to its multiple biological activities and few negative effects. Accumulated studies prove that curcumol shows beneficial effects in protecting against inflammation, oxidative stress, cancer, neurodegeneration and microbial infection [16]. Besides, a number of signaling pathways such as MAPK, PI3K/Akt and NF-κB are influenced by curcumol [17]. As curcumol exhibits inhibitory effects on inflammation and oxidative stress, we speculated that curcumol might show some benefits in cerebral ischemia. In the present study, cerebral ischemia was induced in C57BL/6 mice via MCAO, then treated with curcumol immediately after reperfusion. We found that curcumol treatment ameliorated neuroinflammation and improved stroke outcome. Curcumol skewed microglial polarization and Treg/Th17 balance towards anti-inflammatory phenotype. Besides, curcumol inhibited ROS production and regulated Nrf2/HO-1 and NF-κB signaling in microglia. Our data elucidated a novel role of curcumol in cerebral ischemia.

## Results

### Poststroke administration of curcumol reduces infarct volume and improves motor function recovery of MCAO mice

**Fig. 1:**
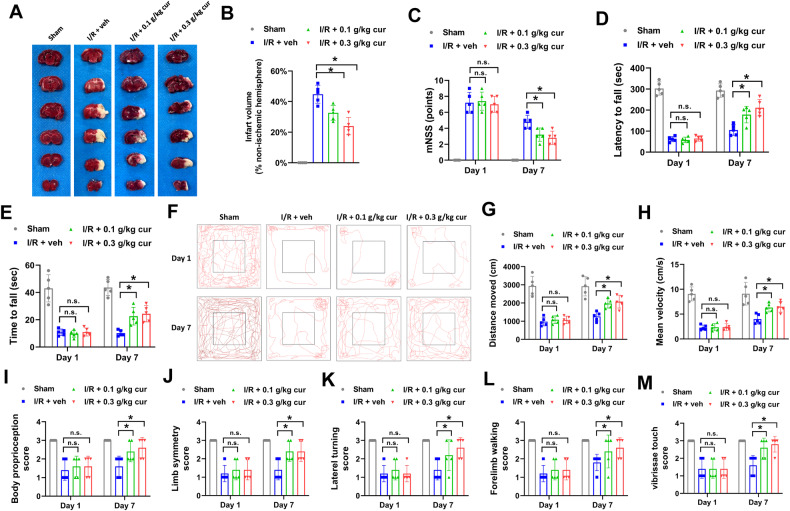
Poststroke administration of curcumol reduces infarct volume and improves motor function recovery in MCAO mice. **A**, **B** MCAO mice were treated with 0.1 g/kg curcumol (I/R + 0.1 g/kg cur group), 0.3 g/kg curcumol (I/R + 0.3 g/kg cur group) and equal volume of PBS (I/R + veh group and Sham group) daily for 7 d post-MCAO. Representative images of TTC staining (**A**) and quantification of infarct volume (**B**) were shown. **C** Neurological deficit score was evaluated by mNSS at day 1 and day 7 after MCAO. D-E, the time of latency to fall (**D**) in rotarod test and time to fall (**E**) in inverted wire mesh grid grip test were shown. **F**–**H** Motion trajectory (**F**), distance moved (**G**), and mean velocity (**H**) of mice were evaluated by open field test at day 1 and day 7 after MCAO. **I**–**M** modified Garcia scores were used to evaluate sensorimotor deficits. The scores for body proprioception (**I**), limb symmetry (**J**), lateral turning (**K**), forelimb walking (**L**), and vibrissae touch (**M**) were shown. *P < 0.05.

To evaluate the influence of curcumol on cerebral ischemia/reperfusion injury and poststroke functional recovery, infarction was induced in mice via MCAO, then treated with different concentrations of curcumol daily for one week immediately after reperfusion. At the end of drug treatment, the infarct volume was evaluated by TTC staining. Compared with MCAO mice in vehicle-treated group (I/R + veh), the infarct volume of MCAO mice in curcumol-treated group (I/R + 0.1 g/kg cur and I/R + 0.3 g/kg cur) was evidently reduced (Fig. 1A, B). In the functional test, MCAO mice had severe motor deficit as evidenced by higher modified neurological severity scores (mNSS) at day 1 poststroke (Fig. 1C). However, the mNSS of mice in curcumol-treated group (I/R + 0.1 g/kg cur and I/R + 0.3 g/kg cur) were apparently decreased compared with mice in vehicle-treated group (I/R + veh) at day 7 poststroke, indicating that curcumol promoted motor function recovery (Fig. 1C). In rotarod test and inverted wire mesh grip test, curcumol treatment increased the strength and motor coordination recovery of MCAO mice as they had longer time to fall at day 7 poststroke (Fig. 1D, E). In open field test, MCAO mice in vehicle or curcumol-treated group had decreased spontaneous exploration behavior, distance moved and mean velocity compared with mice in sham group at day 1 poststroke (Fig. 1F–H). Meanwhile, curcumol treatment obviously increased spontaneous exploration behavior, distance moved and mean velocity of MCAO mice compared with mice in vehicle-treated group at day 7 poststroke (Fig. 1F–H). Next, sensorimotor deficits were evaluated in MCAO mice. Curcumol treatment obviously increased the scores of body proprioception, limb symmetry, lateral turning and forelimb walking of MCAO mice compared with mice in vehicle-treated group at day 7 poststroke (Fig. 1I–L). Taken together, these results indicated that poststroke administration of curcumol reduced infarct volume and improved motor function recovery of MCAO mice.

### Curcumol ameliorate poststroke neuronal damage and neuroinflammation in MCAO mice

**Fig. 2:**
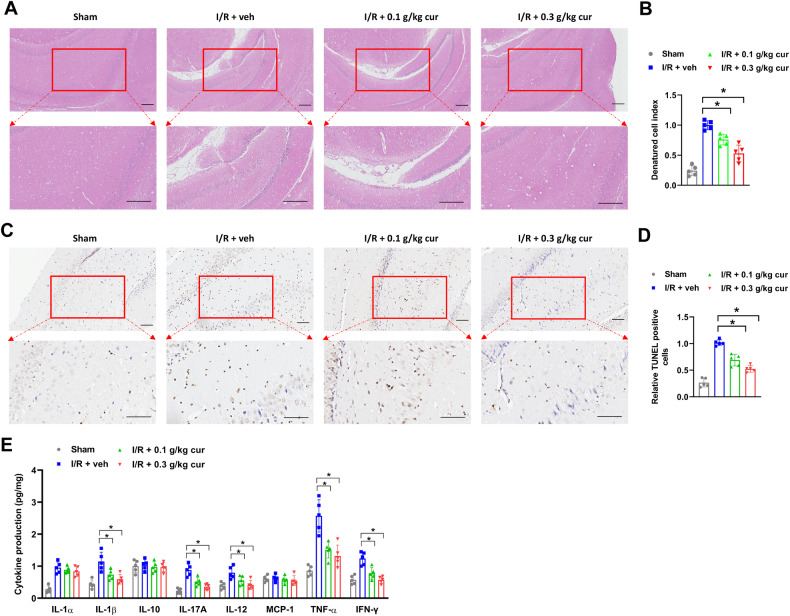
Curcumol ameliorate poststroke neuronal damage and neuroinflammation in MCAO mice. **A**, **B** histopathological changes of brain tissues of MCAO mice were evaluated by H&E staining (**A**). Denatured cell index was shown (**B**). Scale bar = 200 μm. **C**, **D** apoptotic cells in brain tissues of MCAO mice were evaluated by TUNEL staining (**C**). Relative number of TUNEL positive cells were shown (**D**). Arrows indicated TUNEL-positive cells. Scale bar = 100 μm. **E** the levels of pro-inflammatory cytokines in brain tissues of MCAO mice were evaluated by ELISA assay. *P < 0.05.

**Fig. 3:**
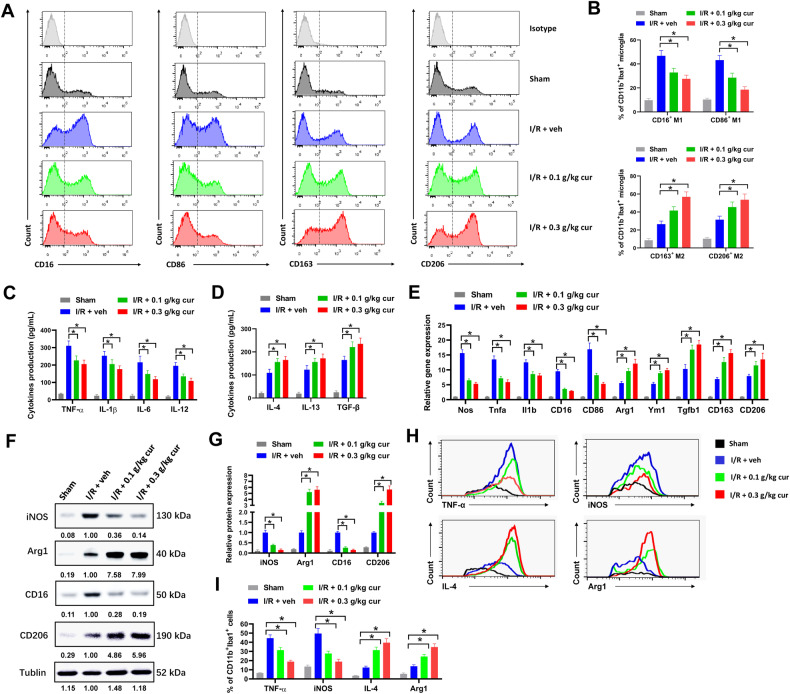
Curcumol skews microglial polarization toward anti-inflammatory phenotype in MCAO mice. **A**, **B** representative histogram (**A**) and percentages of CD16^+^, CD86^+^, CD163^+^ and CD206^+^ cells (**B**) in CD11b^+^Iba1^+^ microglia of MCAO mice were shown. **C**, **D** the levels of proinflammatory cytokines TNF-α, IL-1β, IL-6 and IL-12 (**C**) and anti-inflammatory cytokines IL-4, IL-13 and TGF-β (**D**) in primary microglia of MCAO mice were evaluated by ELISA assay. **E** the mRNA expression of proinflammatory marker genes (Nos, Tnfa, Il1b, CD16 and CD86) and anti-inflammatory marker genes (Arg1, Ym1, Tgfb1, CD163 and CD206) in primary microglia of MCAO mice was evaluated by RT-qPCR. **F**, **G** the protein expression of indicated genes was evaluated by western blot (**F**). Relative protein expression was shown (**G**). **H**, **I** the expression levels of M1 phenotype markers (TNF-α and iNOS) and M2 phenotype markers (IL-4 and Arg1) were detected by flow cytometry. Representative histogram (**H**) and percentages of TNF-α^+^, iNOS^+^, IL-4^+^ and Arg1^+^ cells were shown (**I**). *P < 0.05.

Next, the influence of curcumol on neuronal damage and inflammation in MCAO mice was evaluated. Histopathological changes of brain tissues of MCAO mice were checked by IHC staining. Compared with mice in sham group, MCAO mice in vehicle-treated group (I/R + veh) showed apparently damaged neuron structure, neuronal loss, nuclear condensation, numerous vacuolated spaces and increased denature cell index in brain tissues (Fig. 2A, B). However, curcumol treatment alleviated these pathological changes in brain tissues of MCAO mice compared with vehicle-treated mice (Fig. 2A, B). Neuronal damage was further evaluated by TUNEL staining. The number of TUNEL positive cells in brain tissues of MCAO mice were significantly increased compared with mice in sham group (Fig. 2C, D). Meanwhile, the number of TUNEL positive cells were evidently decreased in curcumol-treated MCAO mice compared with vehicle-treated MCAO mice (Fig. 2C, D). Neuroinflammation was further evaluated by measuring the production of pro-inflammatory cytokines in brain tissues. In our study, pro-inflammatory cytokines such as IL-1β, IL-17A, IL-12, TNF-α and IFN-γ were significantly increased in brain tissues of vehicle-treated MCAO mice compared with mice in sham group (Fig. 3E). In comparison, these cytokines were decreased in curcumol-treated MCAO mice compared with vehicle-treated MCAO mice (Fig. 3E). Collectively, the above results indicated that curcumol ameliorated poststroke neuronal damage and neuroinflammation in MCAO mice.

### Curcumol skews microglial polarization toward anti-inflammatory phenotype in MCAO mice

Microglial polarization plays an important role in neuroinflammation and brain damage of cerebral ischemia [7]. The potential influence of curcumol on microglial polarization was evaluated by flow cytometry. Iba1 and CD11b are widely used surface markers for microglia. CD16 and CD86 are differentiation markers for proinflammatory M1 macrophage. CD163 and CD206 are differentiation markers for anti-inflammatory M2 macrophage. Thus, M1 microglia were marked as CD11b^+^Iba1^+^CD16^+^ or CD11b^+^Iba1^+^CD86^+^, while M2 microglia were marked as CD11b^+^Iba1^+^CD163^+^ or CD11b^+^Iba1^+^CD206^+^ in our study. Curcumol treatment evidently increased the percentage of CD11b^+^Iba1^+^CD163^+^ and CD11b^+^Iba1^+^CD206^+^ M2 microglia and reduced the percentage of CD11b^+^Iba1^+^CD16^+^ and CD11b^+^Iba1^+^CD86^+^ M1 microglia in MCAO mice, suggesting that curcumol promoted anti-inflammatory M2 microglial polarization and suppressed proinflammatory M1 microglial polarization (Fig. 3A, B). Next, the production of proinflammatory cytokines and anti-inflammatory cytokines were evaluated by ELISA assay. We found that curcumol treatment reduced the production of proinflammatory cytokines (TNF-α, IL-1β, IL-6 and IL-12) and increased the production of anti-inflammatory cytokines (IL-4, IL-13 and TGF-β) in primary microglia of MCAO mice (Fig. 3C, D). Microglial phenotypes can be differentiated by the expression of their marker genes. In our study, curcumol treatment evidently decreased the mRNA expression of proinflammatory related genes (Nos, Tnfa, Il1b, CD16 and CD86) and increased the mRNA expression of anti-inflammatory related genes (Arg1, Ym1, Tgfb1, CD163, and CD206) in primary microglia of MCAO mice (Fig. 3E). Some of these genes were further evaluated by western blot. The protein levels of iNOS and CD16 were reduced while Arg1 and CD206 were increased by curcumol treatment in brain tissues of MCAO mice (Fig. 3F, G). The expression levels of M1 phenotype markers (TNF-α and iNOS) and M2 phenotype markers (IL-4 and Arg1) were detected by flow cytometry. The percentages of TNF-α^+^ and iNOS^+^ microglia were apparently reduced while IL-4^+^ and Arg1^+^ microglia increased by by curcumol treatment in brain tissues of MCAO mice (Fig. 3H, I). Above all, these results indicated that curcumol skewed microglial polarization toward anti-inflammatory phenotype in MCAO mice.

### Curcumol induces anti-inflammatory microglial polarization in vitro after OGD/R

To further evaluate the potential influence of curcumol on microglial polarization, an in vitro cell model of cerebral ischemia/reperfusion injury was constructed by exposing primary microglia with OGD/R as previously reported [18]. In our study, curcumol treatment apparently reduced the percentage of CD11b^+^Iba1^+^CD16^+^ and CD11b^+^Iba1^+^CD86^+^ M1 microglia and increased the percentage of CD11b^+^Iba1^+^CD163^+^ and CD11b^+^Iba1^+^CD206^+^ M2 microglia after OGD/R (Supplementary Fig. 1A, B). The expression of M2 microglia marker CD206 was further evaluated by immunofluorescence. Curcumol treatment evidently increased the fluorescence intensity of CD206 and reduced the fluorescence intensity of CD86 in primary microglia after OGD/R (Supplementary Fig. 1C, D). In addition, curcumol treatment suppressed the production of proinflammatory cytokines (TNF-α, IL-1β, IL-6 and IL-12) and promoted the production of anti-inflammatory cytokines (IL-4, IL-13 and TGF-β) in primary microglia after OGD/R (Supplementary Fig. 2A, B). Curcumol treatment also reduced the mRNA expression of proinflammatory marker genes (Nos, Tnfa, Il1b, CD16 and CD86) and promoted the mRNA expression of anti-inflammatory marker genes (Arg1, Ym1, Tgfb1, CD163 and CD206) in primary microglia after OGD/R (Supplementary Fig. 2C). In western blot analysis, curcumol treatment reduced the expression of iNOS and CD16, and increased the expression of Arg1 and CD206 (Supplementary Fig. 2D, E). The expression levels of M1 phenotype markers (TNF-α and iNOS) and M2 phenotype markers (IL-4 and Arg1) were detected by flow cytometry. The percentages of TNF-α^+^ and iNOS^+^ microglia were significantly decreased while IL-4^+^ and Arg1^+^ microglia increased by curcumol treatment after OGD/R (Supplementary Fig. 2F, G). Next, the influence of curcumol on neuron damage was evaluated in vitro. Primary neurons were co-cultured with post-OGD/R microglia in transwell chamber, then apoptotic neurons were evaluated by flow cytometry and TUNEL staining. In flow cytometry, the percentage of Annexin V^+^ apoptotic neurons were reduced when co-cultured with curcumol-treated microglia compared with vehicle-treated microglia (Supplementary Fig. 3A, B). In TUNEL staining, the number of TUNEL positive neurons were decreased when co-cultured with curcumol-treated microglia (Supplementary Fig. 3C, D). Taken together, our results indicated that curcumol induced anti-inflammatory microglial polarization and protected against neuron apoptosis in vitro after OGD/R.

### Curcumol reduces local T cell infiltration in brains of MCAO mice and impairs Treg/Th17 balance

**Fig. 4:**
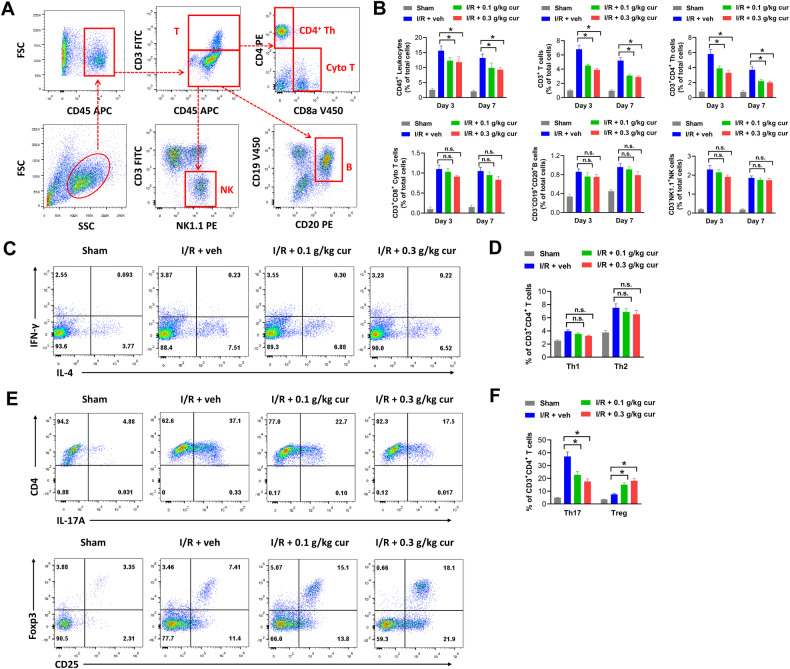
Curcumol reduces local T cell infiltration and impairs Treg/Th17 balance in brains of MCAO mice. **A** representative gating strategies in flow cytometry were shown. **B** the percentages of CD45^+^ Leukocytes, CD3^+^ T cells, CD3^+^CD4^+^ Th cells, CD3^+^CD8^+^ Cyto T cells, CD3^−^CD19^+^CD20^+^ B cells and CD3^−^NK1.1^+^ NK cells in ischemic brain tissues of MCAO mice were evaluated by flow cytometry. **C**–**F** representative histogram (**C**, **E**) and the percentages of IFN-γ^+^IL-4^−^ Th1, IFN-γ^−^IL-4^+^ Th2 cells, IL-17A^+^ Th17 cells and CD25^+^Foxp3^+^ Treg cells (**D**, **F**) in CD3^+^CD4^+^ Th cells of ischemic brain tissues were evaluated by flow cytometry. *P < 0.05.

Activated microglia vividly interact with other leukocytes and potentially influence their infiltration in ischemic brain [19, 20]. Curcumol affected microglial polarization in brain tissues of MCAO mice, thus we speculated that curcumol might also influence the infiltration of other immune cells. Cellular components from the brain tissues of MCAO mice at day 3 and day 7 post-stroke were analyzed by flow cytometry. Representative gating strategies for T cells (CD45^+^CD3^+^), CD4^+^ T helper cells (Th, CD45^+^CD3^+^CD4^+^), CD8^+^ cytotoxic T cells (Cyto T, CD45^+^CD3^+^CD8^+^), Natural killer cells (NK, CD45^+^CD3^−^NK1.1^+^) and B cells (CD45^+^CD3^−^CD19^+^CD20^+^) were shown in Fig. 4A. In our study, CD45^+^ leukocytes were significantly increased in ischemic brain tissues of MCAO mice compared with those in sham group, suggesting enhanced immune infiltration after stroke (Fig. 4B). Meanwhile, curcumol treatment evidently reduced the percentage of infiltrating CD45^+^ leukocytes in ischemic brain compared with that in vehicle-treated mice (Fig. 4B). Moreover, curcumol significantly suppressed the infiltration of CD3^+^ T cells, specifically, CD4^+^ Th cells after stroke (Fig. 4B). In comparison, infiltration of CD8^+^ cytotoxic T, NK and B cells were not affected by curcumol in ischemic brain (Fig. 4B). CD4^+^ Th cells comprise of a number of phenotypically and functionally distinct subsets, including T helper 1 (Th1, IFN-γ^+^IL-4^−^), T helper 2 (Th2, IFN-γ^−^IL-4^+^), T helper 17 (Th17, IL-17A^+^) and regulatory T (Treg, CD25^+^Foxp3^+^). Among them, the percentages of IFN-γ^+^IL-4^−^ Th1 and IFN-γ^−^IL-4^+^ Th2 cells were not significantly affected by curcumol in ischemic brain tissues (Fig. 4C, D). However, cucumol dramatically decreased the percentage of IL-17A^+^ Th17 cells and increased the percentage of CD25^+^Foxp3^+^ Treg cells in ischemic brain tissues of MCAO mice compared with those in vehicle-treated MCAO mice, indicating that curcumol impaired Treg/Th17 balance in ischemic brain (Fig. 4E, F). Collectively, these results indicated that curcumol reduced local T cell infiltration in brains of MCAO mice and impaired Treg/Th17 balance in vivo.

### Curcumol-treated microglia modulate Treg/Th17 balance in vitro

**Fig. 5:**
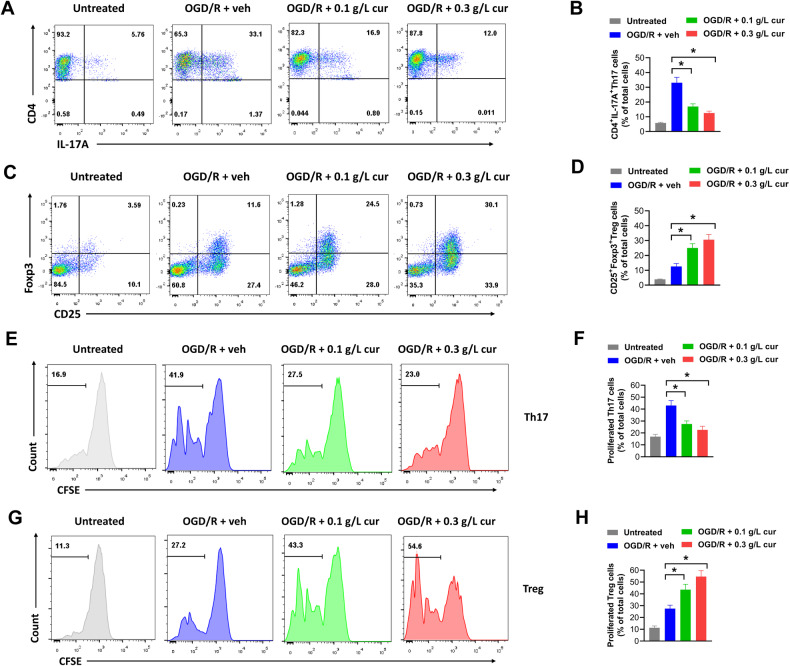
Curcumol-treated microglia modulates Treg/Th17 balance in vitro. Primary microglia were treated with 0.1 g/L or 0.3 g/L curcumol or equal volume of PBS (vehicle) after OGD/R, then co-cultured with CFSE-labelled splenic T cells for 7 days. **A**–**D** representative plots (**A**, **C**) and percentages of CD4^+^IL-17A^+^ Th17 cells and CD25^+^Foxp3^+^ Treg cells (**B**, **D**) in CFSE-labelled splenic T cells were shown. **E**–**H** representative histogram (**E**, **G**) and percentages of CFSE negative proliferative Th17 and Treg cells (**F**, **H**) were shown. *P < 0.05.

Accumulated studies suggest that crosstalk between microglia and Treg/Th17 cells contributes to brain damage and recovery after ischemic stroke [21]. Therefore, we speculated that curcumol-induced microglial polarization might influence Treg/Th17 balance. To evaluate this, primary microglia were treated with curcumol or vehicle after OGD/R, then co-cultured with CFSE-labelled splenic T cells. The percentages of CD4^+^IL-17A^+^ Th17 cells and CD25^+^Foxp3^+^ Treg cells were evaluated by flow cytometry. In our study, co-cultured with curcumol-treated microglia evidently reduced the percentage of CD4^+^IL-17A^+^ Th17 cells and increased the percentage of CD25^+^Foxp3^+^ Treg cells in CFSE-labelled splenic T cells compared with cells co-cultured with vehicle-treated microglia (Fig. 5A–D). Next, proliferation of these Th17 cells and Treg cells were evaluated by checking the percentage of CFSE negative cells. We found that Th17 cells from splenic T cells co-cultured with curcumol-treated microglia were less proliferative compared with these co-cultured vehicle-treated macrophages (Fig. 5E, F). In comparison, Treg cells from splenic T cells co-cultured with curcumol-treated microglia were more proliferative compared with these T cells co-cultured vehicle-treated macrophages (Fig. 5G, H). Above all, these results suggested that curcumol-treated microglia modulated Treg/Th17 balance in vitro.

### Curcumol inhibits ROS production and regulates Nrf2/HO-1 and NF-κB signaling in microglia

**Fig. 6:**
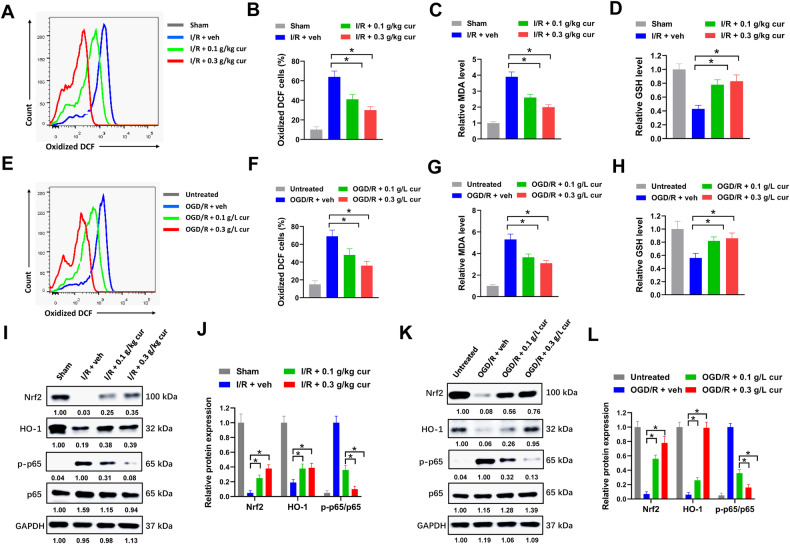
Curcumol inhibits ROS production and Nrf2/HO-1/NF-κB signaling in microglia. **A**, **B** primary microglia from MCAO mice were incubated with DCF-DA, then oxidized DCF was analyzed by flow cytometry. Representative histogram (**A**) and percentages of oxidized DCF cells (**B**) were shown. **C**, **D** relative MDA (**C**) and GSH (**D**) levels in primary microglia from MCAO mice were shown. **E**, **F** primary microglia treated with OGD/R were incubated with DCF-DA, then oxidized DCF was analyzed by flow cytometry. Representative histogram (**E**) and percentages of oxidized DCF cells (**F**) were shown. **G**, **H** relative MDA (**G**) and GSH (**H**) levels of primary microglia treating with OGD/R were shown. **I**–**L** protein levels of indicated genes in primary microglia of MCAO mice or post OGD/R were evaluated by western blot (**I**, **K**). Relative protein levels (**J**, **L**) were shown. *P < 0.05.

There are increasing evidences indicating that oxidative stress is involved in microglial polarization [22]. Oxidative stress sustains M1 phenotype by promoting glycolysis and lipid biosynthesis, and inhibiting tricarboxylic acid cycle and oxidative phosphorylation [23]. DCF-DA is a fluorescent probe to detect cellular ROS production. In our study, primary microglia from MCAO mice were incubated with DCF-DA, then oxidized DCF was analyzed by flow cytometry. We found that curcumol treatment evidently reduced the percentage of oxidized DCF positive microglia compared with vehicle control in MCAO mice, indicating that curcumol suppressed ROS generation in microglia (Fig. 6A, B). Lipid peroxidation was evaluated by measuring MDA levels. We found that the levels of MDA were apparently decreased by curcumol treatment in primary microglia of MCAO mice compared with microglia treated with vehicle control (Fig. 6C). Glutathione (GSH) plays pivotal role in protecting cells against oxidative stress-induced cellular damage. Curcumol treatment significantly increased the level of GSH in primary microglia of MCAO mice (Fig. 6D). This was also tested in curcumol treated primary microglia after OGD/R. The percentages of oxidized DCF positive microglia were significantly reduced by curcumol treatment (Fig. 6E, F). Moreover, curcumol treatment suppressed MDA production and increased GSH levels in primary microglia after OGD/R (Fig. 6G, H). These results suggested that curcumol inhibited ROS production in primary microglia in vivo and in vitro. Nrf2 plays an important role in anti-oxidative stress in cerebral ischemia, while the transcriptional factor NF-κB is a key regulator in inflammation and macrophage polarization [24]. The influence of curcumol on Nrf2 and its downstream target HO-1, and NF-κB signaling in primary microglia was evaluated by western blot. We found that curcumol treatment apparently increased the levels of Nrf2 and HO-1 and reduced the level of phosphorylated NF-κB p65 in primary microglia of MCAO mice or after OGD/R compared with microglia treated with vehicle control (Fig. 6I–L). Taken together, our data indicated that curcumol inhibited ROS production and regulated Nrf2/HO-1 and NF-κB signaling in microglia.

### Inhibiting Nrf2/HO-1 signaling or activating NF-κB signaling abrogates the influence of curcumol on microglial polarization

**Fig. 7:**
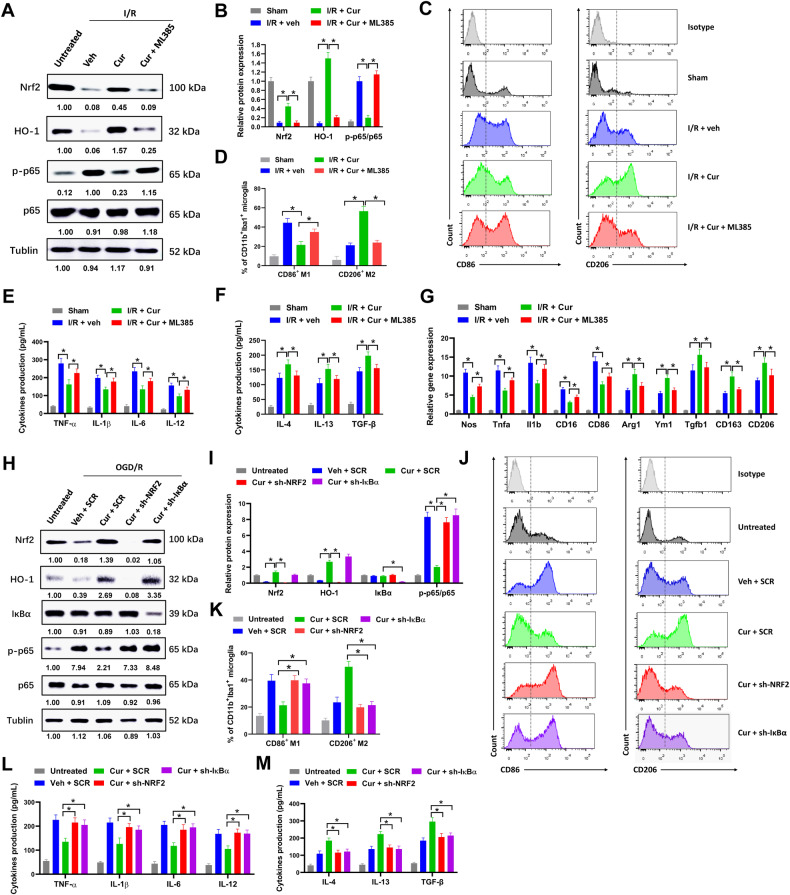
Inhibiting of Nrf2/HO-1/NF-κB signaling abrogates the influence of curcumol on microglial polarization in vivo. Mice were treated with 0.3 g/kg curcumol (cur), 30 mg/kg ML385 or equal volume of DMSO (veh) intraperitoneally for 7 days after MCAO as indicated. **A**, **B** protein levels of indicated genes in primary microglia of MCAO mice were evaluated by western blot (**A**). Relative protein levels (**B**) were shown. **C**, **D** representative histogram (**C**) and percentages of CD86^+^ and CD206^+^ cells (**D**) in CD11b^+^Iba1^+^ microglia of MCAO mice were shown. **E**, **F** the levels of proinflammatory cytokines TNF-α, IL-1β, IL-6 and IL-12 (**E**) and anti-inflammatory cytokines IL-4, IL-13 and TGF-β (**F**) in primary microglia of MCAO mice were evaluated by ELISA assay. **G** the mRNA expression of proinflammatory marker genes (Nos, Tnfa, Il1b, CD16 and CD86) and anti-inflammatory marker genes (Arg1, Ym1, Tgfb1, CD163 and CD206) in primary microglia of MCAO mice was evaluated by RT-qPCR. **H**–**M** post-OGD/R primary microglia were transfected with sh-NRF2, sh-IκBα or SCR as indicated, then treated with 0.3 g/L curcumol (cur) or equal volume of DMSO (veh) for 72 h. Collected cell lysates for western blot (**H**, **I**). Percentages of CD86^+^ and CD206^+^ cells in CD11b^+^Iba1^+^ microglia were evaluated by flow cytometry (**J**, **K**). The levels of proinflammatory cytokines TNF-α, IL-1β, IL-6 and IL-12 (**L**) and anti-inflammatory cytokines IL-4, IL-13 and TGF-β (**M**) were evaluated by ELISA assay. *P < 0.05.

To evaluate the influence of Nrf2/HO-1 and NF-κB signaling on curcumol-induced anti-inflammatory microglial polarization, a Nrf2 inhibitor ML385 was used in our study. Curcumol treatment obviously elevated the levels of Nrf2 and HO-1 and reduced the level of phosphorylated NF-κB p65 in primary microglia of MCAO mice, but co-treated with ML385 largely abolished these effects (Fig. 7A. B). Furthermore, curcumol treatment evidently increased the percentage of CD11b^+^Iba1^+^CD206^+^ M2 microglia and reduced the percentage of CD11b^+^Iba1^+^CD86^+^ M1 microglia in MCAO mice, but co-treated with ML385 abrogated these effects (Fig. 7C, D). ML385 treatment also reversed the influence of curcumol on the production of proinflammatory cytokines (TNF-α, IL-1β, IL-6 and IL-12) and anti-inflammatory cytokines (IL-4, IL-13 and TGF-β), and the expression of proinflammatory marker genes (Nos, Tnfa, Il1b, CD16 and CD86) and anti-inflammatory marker genes (Arg1, Ym1, Tgfb1, CD163 and CD206) in brain tissues of MCAO mice (Fig. 7E–G). The influence of ML385 on curcumol-induced anti-inflammatory microglial polarization was further tested in primary microglia post OGD/R in vitro. Curcumol treatment elevated the percentage of CD11b^+^Iba1^+^CD206^+^ M2 microglia and decreased the percentage of CD11b^+^Iba1^+^CD86^+^ M1 microglia post OGD/R, however this was attenuated by ML385 treatment (Supplementary Fig. 4A, B). Moreover, the effects of curcumol treatment on proinflammatory cytokines and genes and anti-inflammatory cytokines and genes were also abrogated by ML385 treatment post OGD/R (Supplementary Fig. 4C–E). Next, the influence of NRF2 and IκBα knockdown on curcumol regulated microglial polarization was evaluated. IκBα is a negative regulator of NF-κB signaling. In our study, knockdown of NRF2 depleted NRF2 expression and inactivated NRF2/HO-1 signaling, while knockdown of IκBα increased the phosphorylation of p65 and activated NF-κB signaling (Fig. 7H, I). NRF2 or IκBα knockdown reversed the influence of curcumol on NRF2/HO-1 and NF-κB signaling compared with scramble control (SCR) (Fig. 7H, I). Moreover, NRF2 and IκBα knockdown abrogated the influence of curcumol on microglial polarization (Fig. 7J, K) and cytokine production post OGD/R (Fig. 7L, M). The above results suggested that inhibiting of Nrf2/HO-1 or activating NF-κB signaling abrogated the influence of curcumol on microglial polarization in vivo and in vivo.

## Discussion

Curcumol, a pharmacologically active sesquiterpenoid, exhibits protective effects in a variety of diseases. For example, curcumol alleviates cardiac dysfunction, myocardial fibrosis and hypertrophy via inhibiting inflammation and cell apoptosis in an animal model of cardiac remodeling [25]. In non-alcoholic fatty liver disease, curcumol inhibits hepatocyte senescence, iron overload and ferritinophagy through YAP/NCOA4 signaling [26]. In EBV-positive nasopharyngeal carcinoma, curcumol suppresses migration and invasion of nasopharyngeal carcinoma cells through downregulating EBNA1 and targeting nucelolin [27]. In the present study, we found that poststroke administration of curcumol reduces infarct volume, neuronal damage and neuroinflammation, and improves motor function recovery in MCAO mice. Indeed, curcumol shows beneficial effects in neurological disorders. For example, inhibitory glycine receptors (GlyRs) are key targets in the treatment of numerous neurological disorders. Wang et al. find that GlyRs are novel targets for curcumol [28]. Besides, curcumol represses epileptic seizure via allosterically activating GABA(A) receptors [29, 30]. Moreover, accumulated studies prove that curcumol suppresses inflammation in some way. In chronic asthmatic mice, curcumol treatment attenuates airway hyperresponsiveness, airway remodeling, and pulmonary inflammation through inhibiting the activation of Wnt/β-catenin pathway [31]. Curcumol may reduce psoriasis-like inflammation via inhibiting JAK1/STAT3 signaling in keratinocytes [32].

In the present study, curcumol treatment skewed microglial polarization toward anti-inflammatory M2 phenotype in MCAO mice in vivo or post OGD/R in vitro. Microglia are critical brain-specific tissue-resident macrophages that play an important role in brain injury and repair. There are increasing evidences demonstrating that M2-like microglia exhibit protective effect in ischemic stroke. For example, IL-4 administration improves long term functional recovery of MCAO mice via inducing M2 phenotype polarization of microglia/macrophages [33]. Melatonin ameliorates brain damage and improves neurological function recovery after ischemic stroke via promoting anti-inflammatory M2 phenotype polarization [34]. ANXA1 is modified by SUMOylation, then SUMOylated ANXA1 facilitates neurological function recovery via increasing M2 microglial polarization in a mouse model of cerebral ischemia [35]. M2 type microglial polarization also play pivotal roles in controlling overactivated inflammation after stroke [36]. Thus, we speculated that curcumol suppressed neuroinflammation in MCAO mice via facilitating M2 microglial polarization. Nrf2 is one of the most important antioxidant transcription factor during cerebral injury [24]. A number of signaling pathways, such as Keap1, PI3K/Akt, HO-1 and NF-κB signaling, were regulated by Nrf2 [24]. In our study, curcumol treatment inhibited ROS production and regulated Nrf2/HO-1 and NF-κB signaling in microglia, while inhibiting of Nrf2/HO-1 signaling or activating NF-κB signaling abrogated the influence of curcumol on microglial polarization. Accumulated studies indicate that ROS production and NF-κB activation are involved in microglial/macrophage polarization. Indeed, curcumol treatment may influence ROS production and NF-κB signaling activation. For example, Li et al. prove that curcumol treatment inhibits NF-κB and TGF-β signaling in RAW246.7 cells, thus downregulates ROS production and secretion of proinflammation factors [37]. In hepatic fibrosis, curcumol treatment causes HSC-T6 cell death via inhibiting PI3K/NF-κB signaling [38]. In liver sinusoidal endothelial cells, curcumol alleviates chronic liver injury via reducing the production of mitochondrial ROS and suppressing of KLF5-mediated liver angiogenesis [39].

The morphology of microglia is highly dynamic and influenced by environmental cues, and efficient remodeling of their structure is a prerequisite to all microglial activities [40]. During M1 type microglial polarization, there are studies documented that in response to injury or damage, microglia undergo somal enlargement, adopt an amoeboid appearance and often exhibit surface expression of CD68 and MHC-II [41]. In contrast, less information is known to the structural changes of M2 microglia, but several culture-based studies indicate that IL-4 and IL-10 can trigger amoeboid microglia undergo significant ramification [42]. In our study, curcumol shifted M1 microglial polarization toward anti-inflammatory M2 phenotype post OGD/R in vitro. However, we did not observe significant morphology changes of microglia during these processes. This is a potential limitation of our study. Possible explanation is that the microglia morphology is highly dynamic and evidently affected by environmental signals, and maybe the morphology changes of microglia are not so apparent in our cell culture system.

In the present study, curcumol treatment reduced local T cell infiltration in brains of MCAO mice and impairs Treg/Th17 balance. Actually, more and more studies reveal that Treg and Th17 cells take part in brain injury and repair after stroke. The number of Tregs are increased in blood and infarct area of ischemic stroke patients [43]. Liesz et al. prove that Tregs are key cerebroprotective immunomodulators in experimental brain ischemia, and depletion of Tregs promotes the activation of resident microglia and invading of T cells [13]. Lee et al. demonstrate that reduced Tregs infiltration by CXCL14 depletion increases inflammation and brain injury in an in vivo ischemia/reperfusion brain model [44]. Moreover, Zhang et al. find that in vivo expansion of Tregs by IL-2/IL-2 antibody complex reduces infarct volume, inhibits neuroinflammation, and improves sensorimotor functions of MCAO mice [45]. Th17 cells are also upregulated in blood and infarct area of ischemic stroke patients [46]. In addition, depletion of Th17 cells by IL-17A knockout ameliorates neuroinflammation and improves stroke outcome in MCAO mice [14]. In a mouse model of ischemic stroke, PR-957 treatment reduces infarct volume and neurological deficits via inhibiting T cell infiltration and Th17 differentiation [47]. In our study, we found that curcumol treatment dramatically reduced the percentage of Th17 cells and increased the percentage of Treg cells in ischemic brain tissues of MCAO mice, thus suppressed neuroinflammation and improved stroke outcome and brain functional recovery. Furthermore, curcumol-treated microglia promoted Treg differentiation and inhibited Th17 differentiation of splenic T cells, indicating that M2 type microglial polarization might shift Treg/Th17 balance towards Treg bias. Indeed, microglial/macrophage polarization and activation may affect T cell infiltration and differentiation. In tenascin-C deficient mice, reduced microglia surveillance promotes T cell infiltration in the ischemic brain parenchyma [48]. In helminth infection and allergy, RELMα expression M2 macrophages promote proliferation and activation of Tregs [49]. In transient insult-induced brain injury, microglia drive the infiltration and clonal expansion of CD8^+^ T lymphocytes via CCL2/CCL8 chemokines [50].

In summary, we found that curcumol reduced infarct volume, attenuated neuronal damage and neuroinflammation, and improved motor function recovery in MCAO mice. Curcumol skewed microglial polarization toward anti-inflammatory M2 phenotype in MCAO mice in vivo and post OGD/R in vitro. Moreover, curcumol reduced local T cell infiltration in brains of MCAO mice and impaired Treg/Th17 balance. Curcumol-treated microglia modulated Treg/Th17 balance in vitro. Finally, curcumol suppressed ROS production and regulated Nrf2/HO-1 and NF-κB signaling in microglia, while inhibiting Nrf2/HO-1 signaling or activating NF-κB signaling abrogated the influence of curcumol on microglial polarization. Our data elucidated a novel protective role of curcumol in cerebral ischemia/reperfusion injury. Curcumol might be a promising therapeutic strategy for stroke.

## Materials and methods

### Transient middle cerebral artery occlusion (MCAO) model

Six-weeks old male C57BL/6 J mice (18–20 g) were purchased from Beijing River Laboratory Animal Corp. Ltd. Mice were kept in constant room temperature (22 ± 2°C), 50 ± 10% humidity and 12/12 light/dark cycle, with free access to water and food. All procedures for animal experiments were reviewed and approved by the Animal Care and Use Committee guidelines of Second Hospital of Hebei Medical University. Focal cerebral ischemia was induced in C57BL/6 J mice via transient occlusion of the middle cerebral artery as previously reported [47]. Briefly, mice were anesthetized by pentobarbital sodium and kept warm on a temperature-regulated heating pad during surgery. Then, made incisions to expose the left common carotid artery (CCA), external carotid artery (ECA) and internal carotid artery (ICA). ECA was ligated at two positions by surgical nylon monofilament near the distal end of CCA. After that, a silicon-coated monofilament was inserted from the left ECA, through the bifurcation of CCA, into the intracranial segment of ICA. The blood flow of middle cerebral artery (MCA) was blocked for 60 min. The regional cerebral blood flow (rCBF) of MCA was evaluated by a laser Doppler flowmeter (Perimed AB, China). A decline of rCBF ≥ 70% was considered as successful occlusion. Then, the silicon-coated monofilament was withdrawn to allow reperfusion. Sham group received the same anesthesia and surgical procedure, except that no filament was inserted into the ICA. After MCAO, 15 mice were randomly divided into I/R + veh group, I/R + 0.1 g/kg cur group and I/R + 0.3 g/kg cur group (5 mice in each group). Sham group had 5 mice. Curcumol was purchased from Selleck chemicals (#S2407) and dissolved in phosphate buffer solution (PBS). Mice were intraperitoneally injected with 0.1 g/kg curcumol (I/R + 0.1 g/kg cur group), 0.3 g/kg curcumol (I/R + 0.3 g/kg cur group) or equal volume of PBS (I/R + veh group and Sham group) daily for 7 d post-MCAO. The NRF2 inhibitor ML385 was purchased from Selleck chemicals (#S8790) and dissolved in DMSO. MCAO mice were treated with 0.3 g/kg curcumol (cur), 30 mg/kg ML385 or equal volume of DMSO (veh) intraperitoneally for 7 days after MCAO as indicated.

### Primary microglia isolation and OGD/R procedure

Primary microglia from adult healthy mice were isolated as previously described [35]. In brief, mice were decapitated, then skull cap was removed to expose and extract the brain. Brain tissues were homogenized and passed through 70 μm nylon cell strainer. The homogenate was centrifuged at 600 × *g* for 5 min, then pellet was resuspended in 70% stock isotonic percoll. Cells were separated by percoll gradient (0%–70%) at 2000 × *g* for 20 min. Microglia from the interface between 50% and 70% stock isotonic percoll were collected in separate conical tubes. These cells were cultured by DMEM/F12 medium (Gibco, USA) supplemented with 10% fetal bovine serum (FBS, Gibco, USA) and 1% Penicillin/Streptomycin (ThermoFisher, USA) in a humidified incubator at 37 °C with 5% CO_2_. Then, isolated cells were stained with anti-mouse Iba-1 FITC (eBioscience, USA) and anti-mouse CD11b APC (eBioscience, USA), and sorted by flow cytometry. The in vitro cerebral ischemia/reperfusion injury model was constructed by exposing primary microglia to OGD/R conditions as previously reported [35]. In brief, microglia were rinsed for two times, then incubated with prewarmed glucose-free DMEM/F12 (Gibco, USA) in an anaerobic incubator containing 5% CO_2_ and 95% N_2_ at 37 °C for 1 h. Then, culture medium was changed to normal DMEM/F12 containing glucose. Cells were cultured under normoxic condition for another 24 h.

### TTC (2,3,5‑triphenyltetrazolium chloride) staining

One week after MCAO surgery, the mice were anesthetized by pentobarbital sodium and perfused with 15 mL cold phosphate-buffered saline (PBS) through the left ventricle. The brain was carefully removed and cut into five coronal blocks (2 mm thick) from the rostral tip of the cerebrum to the caudal tip. The brain slices were stained with 2% TTC at 37 °C for 30 min and fixed by 4% paraformaldehyde at room temperature for 30 min. The white infarct area was calculated by Image J software (NIH, USA). The infarct volume (%) was measured by the equation: (contralateral hemisphere−ipsilateral noninfarct area)/ contralateral hemisphere × 100%.

### Neurological score

The neurological deficit level was assessed by the modified neurological severity score (mNSS) as previously described [18]. Briefly, motor tests (including flexion of forelimb, flexion of hindlimb, and head movement, scored 0–6), beam balance tests (scored 0–6), and reflex and balance tests (score 0–2) were performed at day 1 and day 7 after MCAO. Failure to pass one of these tests will get 1 point. A total score of 1 to 4, 5 to 9, and 10 to 14 represented mild, moderate, and severe neurological dysfunctions. The neurological score was evaluated by three independent researchers.

### Rotarod test and Inverted wire mesh grid grip test

Mice were pretrained 4 times daily for 3 consecutive days using an accelerated rotarod training paradigm in rotarod test. The tests were performed at day 1 and day 7 after MCAO. Before tests, mice were habituated to environment for 30 min. Then, mice were pretrained for 30 min on the rotarod with a speed accelerated from 5 to 10 rpm. Thereafter, the speed of rotarod accelerated from 5 to 40 rpm in 5 min. The latency to fall was record automatically. The procedure was conducted for four consecutive times with a 30 min rest between each test. In inverted wire mesh grid grip test, mice were placed on a metal wire mesh grip and rotated slowly at a steady speed in inverted position. The time to fall from the mesh was recorded. The tests were repeated for 3 times with a 5 min interval.

### Open field test and modified Garcia score test

In open filed test, mice were placed in a plastic test box (40 × 40 × 50 cm) and recorded their free movement for 5 min. The distance moved and mean velocity were record by an automatic video and analyzed. The modified Garcia score test was performed as previously described [51]. The scores for body proprioception, limb symmetry, lateral turning, forelimb walking and vibrissae touch were evaluated at day 1 and day 7 after MCAO.

### Immunohistochemistry (IHC) staining

Mice brains were fixed by 10% formalin, dehydrated, embedded in paraffin and cut into slices (5 μm). Brain slices were then deparaffinized in xylenes and rehydrated by alcohol gradients. Citrate buffer was used for antigen retrieval. Hydrogen peroxide was used to block endogenous peroxidase activity. Then, sections were stained with hematoxylin and eosin (H&E) (Beyotime, China) to evaluate neuronal damage. Neuronal damage was shown as the denatured cell index: degenerated cells/ total number of cells.

### TUNEL staining

Cell apoptosis was evaluated by TUNEL assay kit (Promega, USA) as protocol described. Briefly, brain sections or culture cells were fixed by 4% paraformaldehyde for 15 min at room temperature. Then, sections or cells were incubated with terminal deoxynucleotidyl transferase enzyme for 1 h at room temperature in dark room. Sections or cells were washed with PBS for three times and stained with DAPI for 10 min at room temperature. Images were obtained by a high-resolution digital camera (Microfire, Olympus, USA).

### Flow cytometry

Immune cells infiltrating the ischemic brain were evaluated by flow cytometry. In brief, mice were decapitated, then skull cap was removed to expose and extract the brain. Brain tissues were homogenized and passed through 70 μm nylon cell strainer. The homogenate was centrifuged at 600 × *g* for 5 min, then pellet was resuspended in 70% stock isotonic percoll. Cells were separated by percoll gradient (0%–70%) at 2000 × *g* for 20 min. The supernatant and myelin fraction were removed carefully. Then residue percoll was washed away. Cell fraction was resuspended in PBS containing 2% fetal bovine serum. To discriminate the immune cell populations, cells were stained with antibodies against mouse CD45 APC, CD3 FITC, NK1.1 PE, CD4 PE, CD8a V450, CD19 V450, CD20 PE, CD25 PE-CY7, FoxP3 APC-CY7, CD16 PE, CD86 FITC, CD163 PE, CD206 FITC, IL-4 PE-CY7, IFN-γ APC-CY7 and IL-17A PE-CY7 (all from eBioscience, USA) as indicated. For intracellular staining, cells were firstly fixed and permeabilized using the Fixation/Permeabilization Solution Kit (BD bioscience, USA) according to manufacturers’ instructions. Apoptotic cells were evaluated by staining with Annexin V FITC and PI PE. Cells were evaluated by a FACS AriaTM flow cytometer (BD Biosciences, USA). Data were analyzed by FlowJo v10 software.

### Enzyme-linked immunosorbent assay (ELISA)

The expression levels of IL-1α, IL-1β, IL-4, IL-6, IL-10, IL-12, IL-13, IL-17A, MCP-1, TNF-α, TGF-β and IFN-γ were detected via ELISA assay kits (DAKEWEI, China) according to manufacturers’ instructions. Briefly, samples were incubated with antibody-coated plates for 1 h at room temperature, then washed for 5 times. Next, the plates were incubated with biotinylated antibodies, streptavidin antibodies, substrate solution and stop solution consecutively. Each sample has three duplicates. The optical density at 450 nm was measured by a microplate reader.

### Reverse transcription–quantitative PCR (RT-qPCR)

Total RNAs were extracted by TRIzol reagent (Takara, Japan) as protocol described. Complementary DNA (cDNA) was reverse transcribed using the RevertAid First Strand cDNA Synthesis Kit (ThermoFisher, USA). RT-qPCR was conducted by the SYBR green PCR Master Mix (Roche, Switzerland) on an Opticon 2 real-time PCR detection system (Bio-Rad, USA). The primers used in RT-qPCR were listed in Table 1.

**Table 1:** Primers used in RT-qPCR.

Gene name	Forward primer	Reverse primer
Tnfa	ACCACCATCAAGGACTCA	AGGTCTGAAGGTAGGAAGG
Il11b	GAATCTATACCTGTCCTGTGTA	CTTGTGCTCTGCTTGTGA
Nos	TACTGCTGGTGGTGACAA	CTGAAGGTGTGGTTGAGTT
CD16	TTAGGAGGCTGAGGAAGG	GGCAGTAGGCATCTTGTT
CD86	ATACAGGTGACAGCAACAG	CAGGCAGGTGGATTCTTC
Arg1	AAGGTCTCTACATCACAGAAG	CGAAGCAAGCCAAGGTTA
Tgfb1	CAACAACGCCATCTATGAG	CAAGGTAACGCCAGGAAT
CD163	CACTGGAACTGAGGAACAT	TGGACTGGAGACAATGAAC
CD206	GAGGACTGCGTGGTTATG	TGAAGGTGGATAGAGTGGAT
Ym1	CAGTCAGTTATCAGATTCCTTC	CTCCACAGATTCTTCCTCAA
GAPDH	TCTCCTGCGACTTCAACA	TGTAGCCGTATTCATTGTCA

### Western blot

Protein samples were prepared by RIPA lysis buffer (Beyotime, China) supplemented with protease inhibitors (Sigma, USA). Protein concentration was measured by Bradford reagent (Sigma, USA). A total of 20 μg protein was loaded on SDS-PAGE (8%-12%) and transferred to PVDF membrane (Millipore, USA). Next, the membrane was blocked by 5% non-fat milk and incubated with appropriate first antibody and corresponding second antibody. The western bands were revealed by ECL reagents (ThermoFisher, USA). The antibodies used were: iNOS Rabbit mAb #13120, Arginase-1 Rabbit mAb #93668, CD16/CD32#88280, CD206 Rabbit mAb #24595, GAPDH Rabbit mAb#92310, NRF2 Rabbit mAb #12721, HO-1 Rabbit mAb #43966, NF-κB p65 Rabbit mAb #8242, Phospho-NF-κB p65 (Ser536) Rabbit mAb #3033, β-Tubulin Rabbit mAb #2128 and anti-rabbit IgG, HRP-linked Antibody #7074 from Cell signaling technology (USA). All original western blot gels were upload in the supplementary files.

### Immunofluorescence staining

Cells were seeded on coverslips, then fixed by 4% paraformaldehyde for 10 min and permeabilized by 0.5% Triton X-100 for 5 min at room temperature. Next, the coverslips were blocked by 1% BSA for 15 min at room temperature and incubated with CD206 Rabbit mAb (Cell signaling technology #24595, 1: 50) or CD86 (Abcam #ab119857, 1: 50) at 4°C overnight. Cells were washed for 5 times and incubated with corresponding secondary antibody for 1 h at room temperature. Images were obtained by Olympus Fluorview-3000 confocal microscope (Olympus Optical, Japan).

### T cell proliferation assays

5 × 10^5^ CD3^+^ splenic T cells were stimulated with Dynabeads mouse T-activator CD3/CD28 beads (Life Technologies, USA) for 24 h in 24-well plates. Then, these T cells were incubated with 5×10^5^ microglia in the presence of IL-2 (R&D, USA) for 7 d. Next, cells were used for staining of CD3, CD4, CD25, FoxP3, IL-17A and CFSE, and analyzed by flow cytometry.

### Measurement of ROS, MDA and GSH levels

Cellular ROS were evaluated using the DCF-DA probe. Briefly, microglia (2 × 10^5^) were seeded in 6-well plates and treated as indicated. Then, cells were incubated with 10 μM DCF-DA at 37 °C for 30 min in dark room. Cells were then analyzed by flow cytometry. Malondialdehyde (MDA) and GSH levels were measured by the lipid peroxidation MDA assay kit (Nanjing Jiancheng#A003-4-1, China) and GSH assay kit (Nanjing Jiancheng#A006-2-1, China) according to manufacturers’ instructions.

### Plasmid constructs

To deplete NRF2 or IκBα expression in microglia, short hairpin RNAs targeting mouse NRF2 (sh-NFR2, 5’-GCAGA ATTAT AGCCA AAGCTA-3’) or IκBα (sh-IκBα, 5’-CACTT GGCAA TCATC CACGAA-3’) were cloned into the pLKO.1 plasmid. The pLKO.1 plasmid inserted with a non-targeting sequence was used as scramble control (SCR, 5’-ACGGA GGCTA AGCGT CGCAA-3’).

### Statistical analysis

Data were analyzed by GraphPad Prism 8.0 (GraphPad Software, USA). Difference between groups was evaluated by one-way ANOVA (Tukey’s post-hoc test) or Student’s *t* test. *P* < 0.05 was considered as statistically significant.

### Supplementary information

Supplementary files

## Data Availability

The datasets used and/or analyzed during the current study are available from the corresponding author on reasonable request.
