# Supplementary material for: Curcumol ameliorates neuroinflammation after cerebral ischemia–reperfusion injury via affecting microglial polarization and Treg/Th17 balance through Nrf2/HO-1 and NF-κB signaling
Source: Cell Death Discov. 2024 Jun 24;10:300. doi: 10.1038/s41420-024-02067-3 (PMC11196256; doi:10.1038/s41420-024-02067-3)

Supplementary data

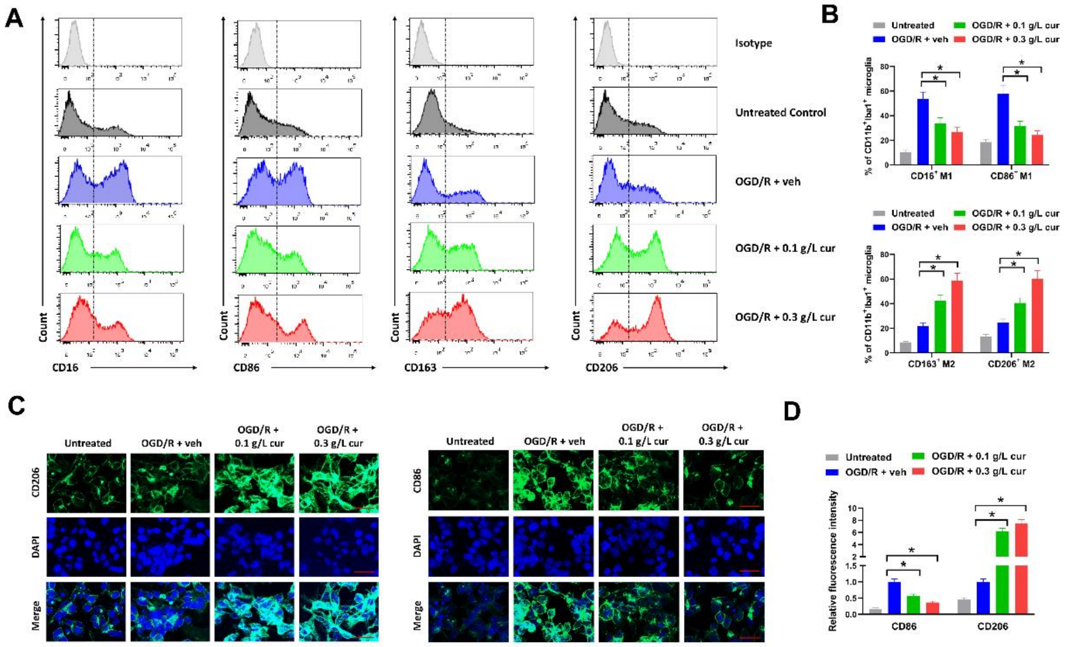

Supplementary Figure 1. Curcumol induces anti-inflammatory microglial polarization *in vitro* post OGD/R. Primary microglia from healthy mice were treated with 0.1g/L curcumol (OGD/R + 0.1g/L cur group), 0.3g/L curcumol (OGD/R + 0.3g/L cur group) and equal volume of PBS (OGD/R + veh group and Sham group) for 72h after OGD/R. A-B, representative histogram (A) and percentages of $CD16^+$, $CD86^+$, $CD163^+$ and $CD206^+$ cells (B) in $CD11b^+Iba1^+$ microglia of primary microglia after OGD/R were evaluated by flow cytometry and shown. C-D, the expression levels of CD86 and CD206 were evaluated by immunofluorescence (C). Relative fluorescence intensity was shown (D). Scale bar = 10 μm. *P <0.05.

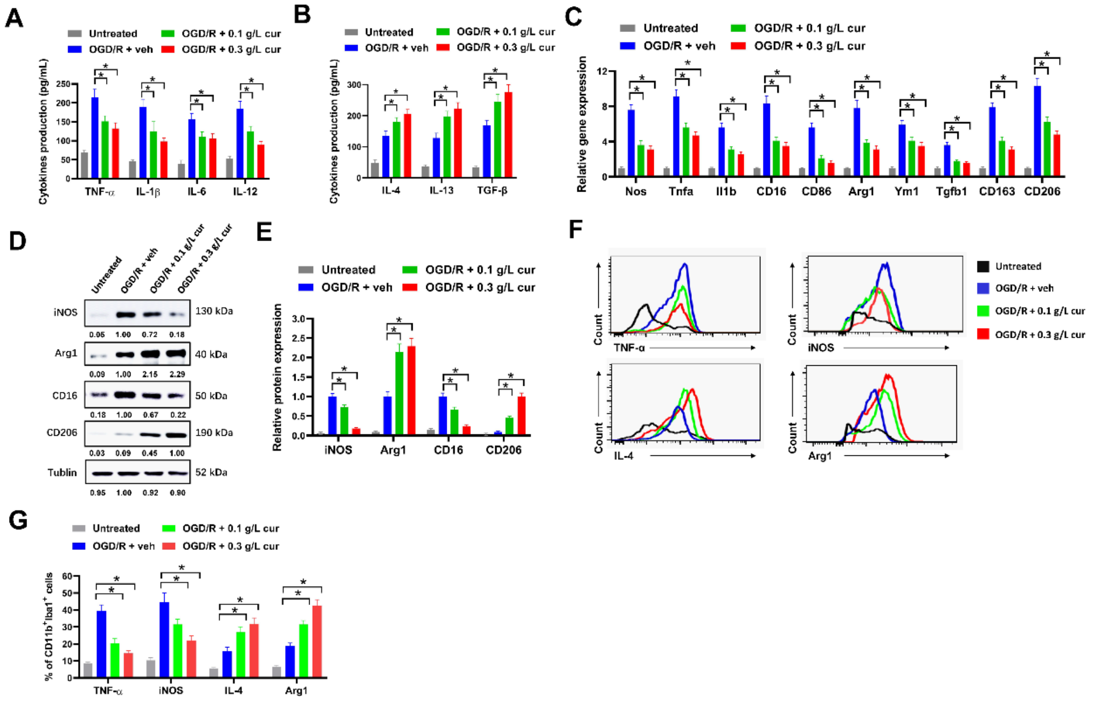

Supplementary Figure 2. Curcumol promotes M1 to M2 microglial polarization *in vitro* post OGD/R. A-B, the levels of proinflammatory cytokines TNF-α, IL-1β, IL-6 and IL-12 (A) and anti-inflammatory cytokines IL-4, IL-13 and TGF-β (B) in primary microglia after OGD/R were evaluated by ELISA assay. C, the mRNA expression of proinflammatory marker genes (Nos, Tnfa, Il1b, CD16 and CD86) and anti-inflammatory marker genes (Arg1, Ym1, Tgfb1, CD163 and CD206) in primary microglia after OGD/R was evaluated by RT-qPCR. D-E, the protein levels of indicated genes in primary microglia after OGD/R were evaluated by western blot (D). Relative protein expression was shown (E). F-G, the expression levels of M1 phenotype markers (TNF-α and iNOS) and M2 phenotype markers (IL-4 and Arg1) were detected by flow cytometry. Representative histogram (F) and percentages of TNF-α$^+$, iNOS$^+$, IL-4$^+$ and Arg1$^+$ cells were shown (G). *P <0.05.

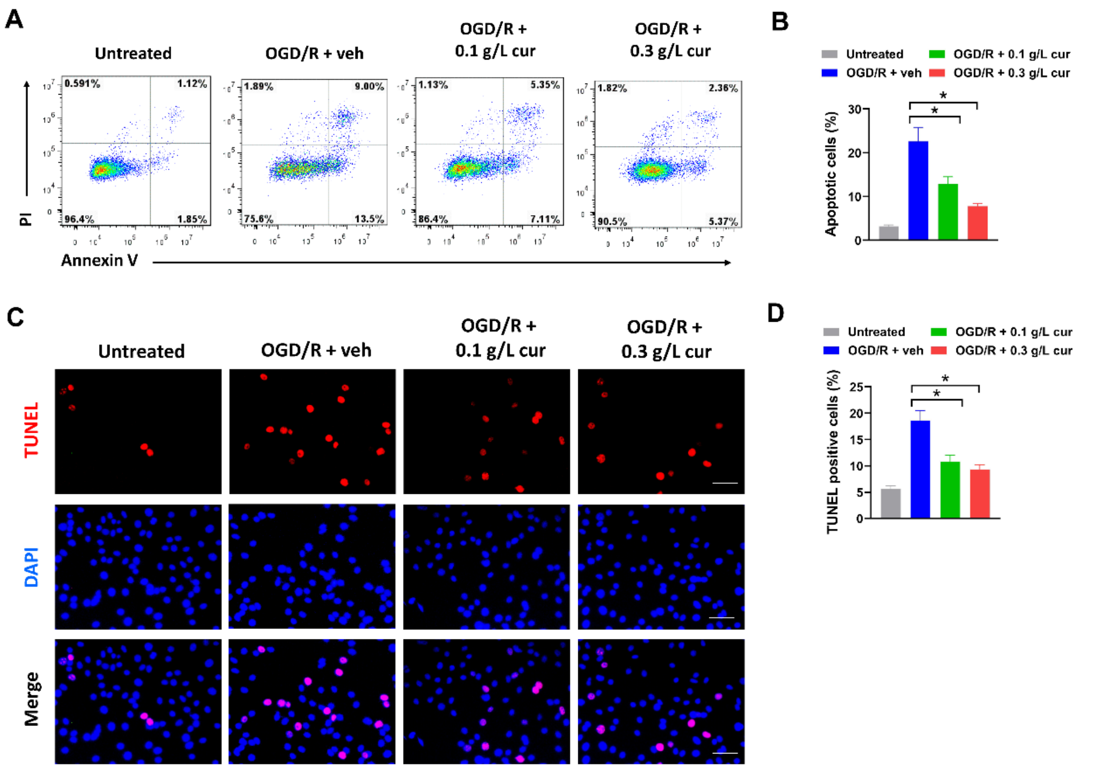

Supplementary Figure 3. Curcumol suppresses microglia-induced neuronal apoptosis post OGD/R. Primary neurons were co-cultured with post-OGD/R primary microglia in transwell chamber for 72 h. Apoptotic cells were evaluated by flow cytometry (A-B) or TUNEL staining (C-D). *P <0.05.

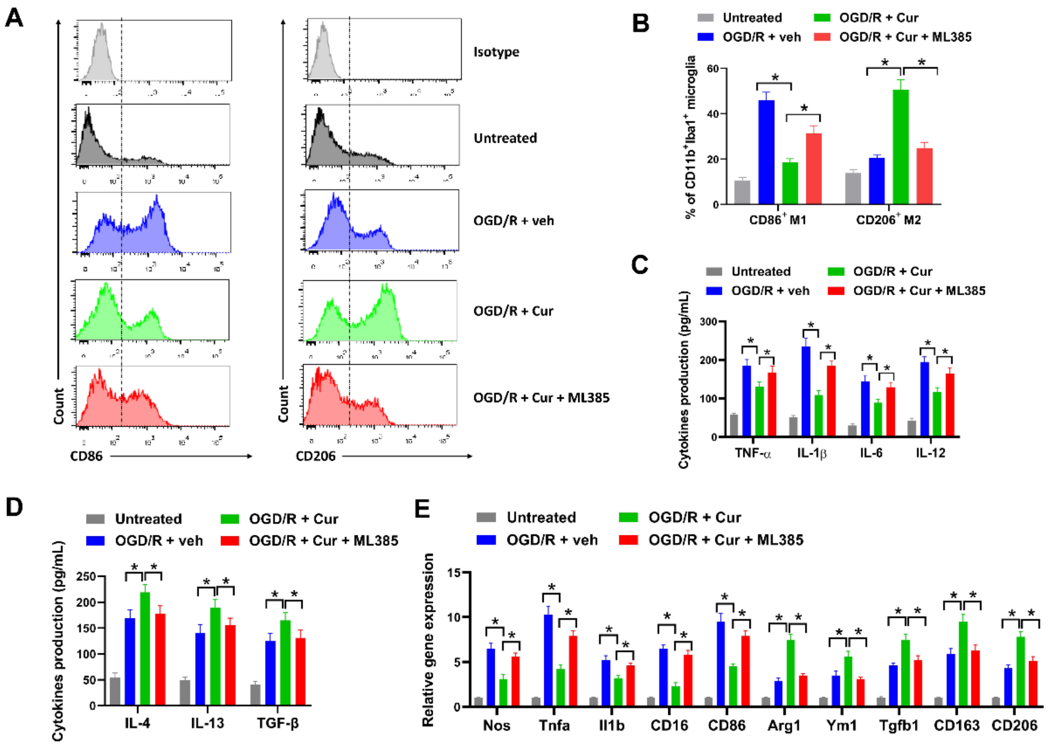

Supplementary Figure 4. ML385 treatment abolishes the effect of curcumol on microglial polarization in vitro. Post-OGD/R primary microglia were treated with 0.3g/L curcumol (cur), 1 μM ML385 or equal volume of DMSO (veh) for 72 h as indicated. A-B, representative histogram (A) and percentages of $CD86^+$ and $CD206^+$ cells (B) in $CD11b^+Iba1^+$ microglia were shown. C-D, the levels of proinflammatory cytokines TNF-α, IL-1β, IL-6 and IL-12 (C) and anti-inflammatory cytokines IL-4, IL-13 and TGF-β (D) were evaluated by ELISA assay. E, the mRNA expression of proinflammatory marker genes (Nos, Tnfa, Il1b, CD16 and CD86) and anti-inflammatory marker genes (Arg1, Ym1, Tgfb1, CD163 and CD206) was evaluated by RT-qPCR. *P <0.05.

Original western blot gels

Fig 3F

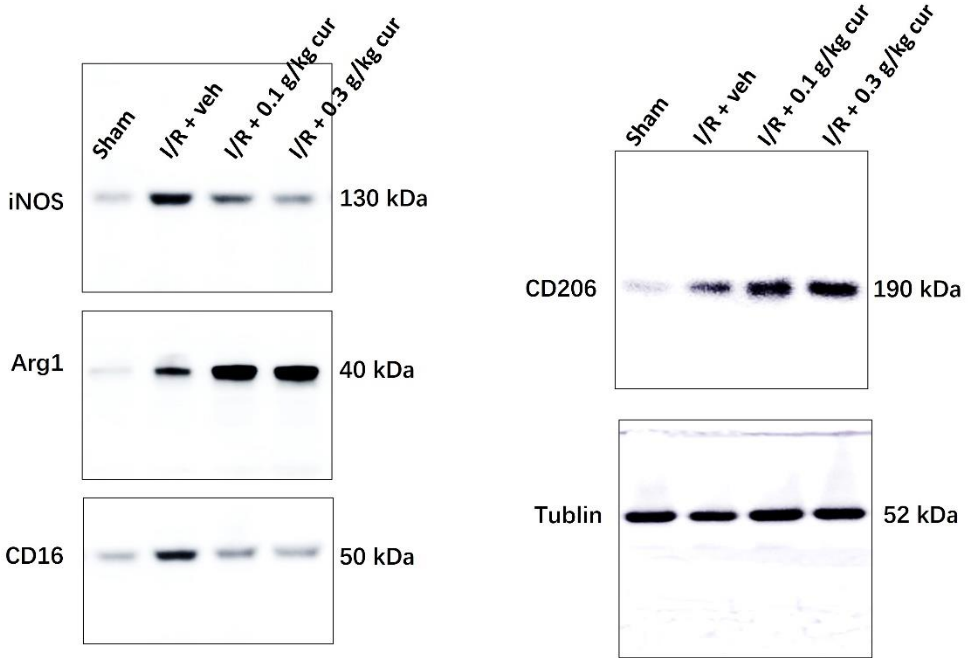

Fig 6I

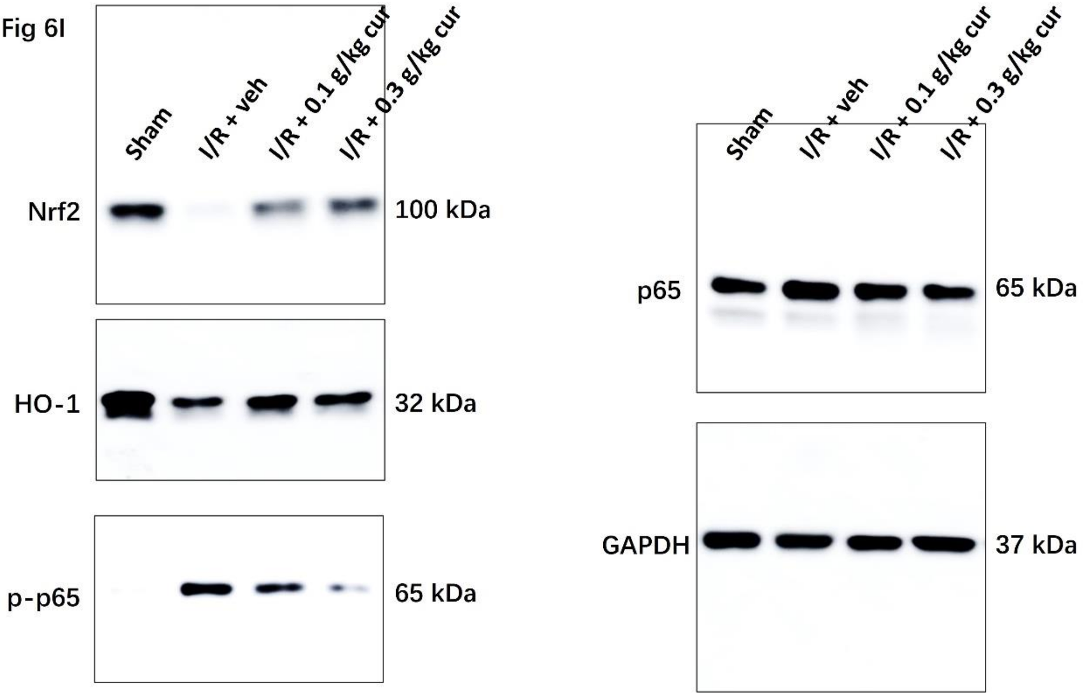

Fig 6K

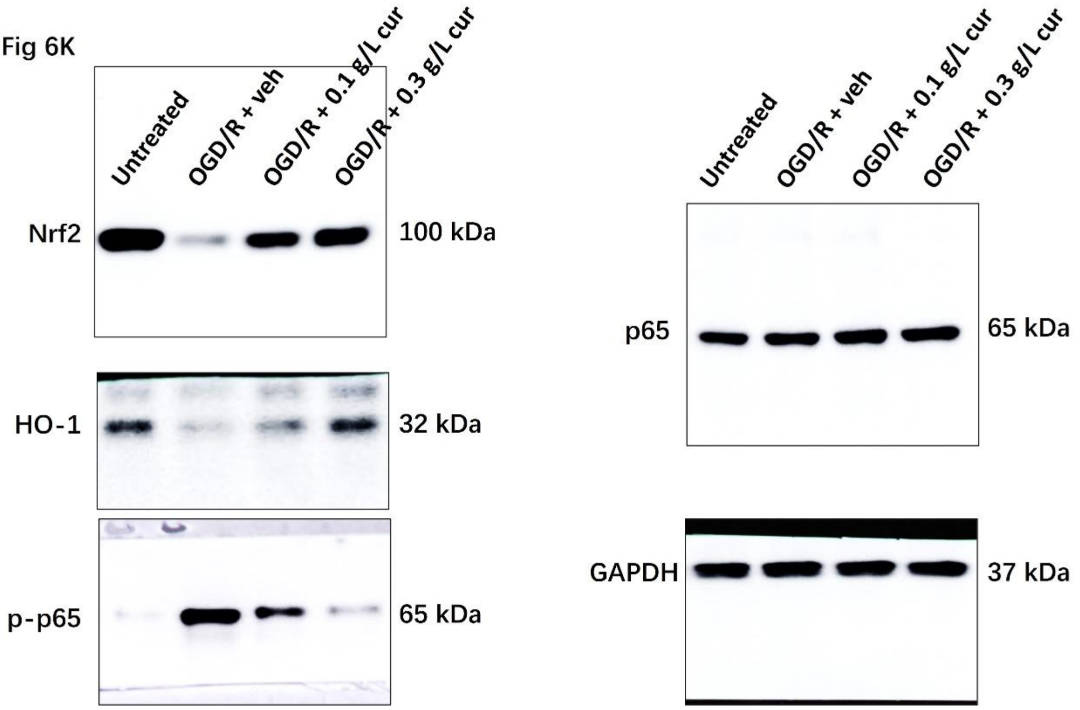

Figure 7A

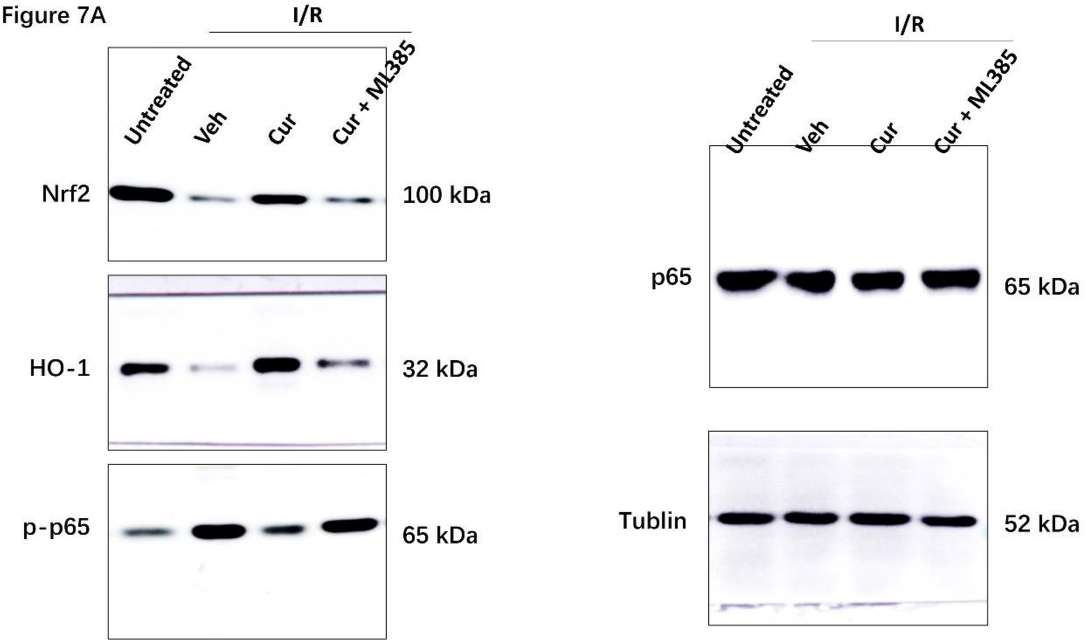

Fig 7H

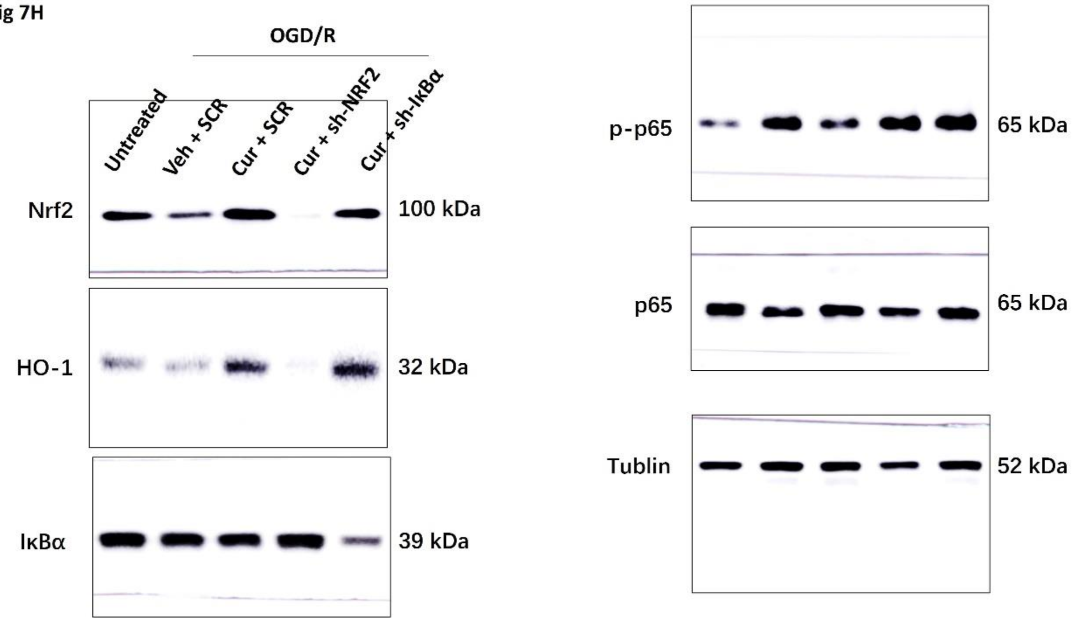

Fig S2D

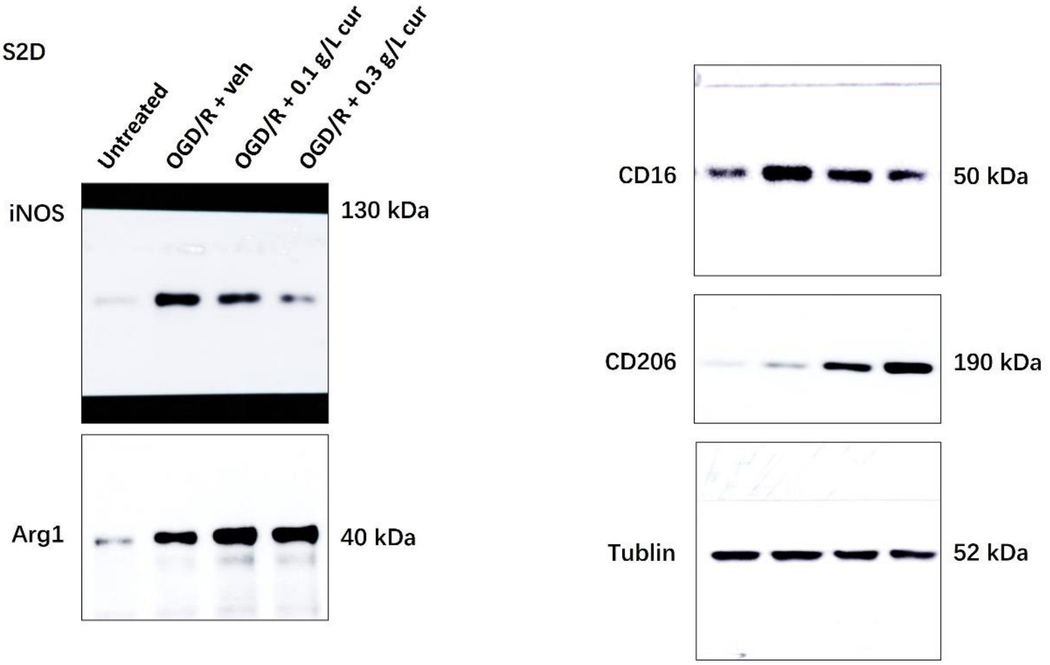

Supplement: Supplementary file 1 — Supplementary files [file 41420_2024_2067_MOESM1_ESM.pdf]
